# Nanocomposites of PCL and SBA-15 Particles Prepared by Extrusion: Structural Characteristics, Confinement of PCL Chains within SBA-15 Nanometric Channels and Mechanical Behavior

**DOI:** 10.3390/polym14010129

**Published:** 2021-12-30

**Authors:** Tamara M. Díez-Rodríguez, Enrique Blázquez-Blázquez, Nadine L. C. Antunes, M. Rosário Ribeiro, Ernesto Pérez, María L. Cerrada

**Affiliations:** 1 Instituto de Ciencia y Tecnología de Polímeros (ICTP-CSIC), Juan de la Cierva 3, 28006 Madrid, Spain; t.diez@ictp.csic.es (T.M.D.-R.); enrique.blazquez@csic.es (E.B.-B.); ernestop@ictp.csic.es (E.P.); 2 Centro de Química Estrutural, Instituto Superior Técnico, Universidade de Lisboa, Av. Rovisco Pais 1, 1049-001 Lisboa, Portugal; nadine.leonor10@gmail.com (N.L.C.A.); rosario@tecnico.ulisboa.pt (M.R.R.)

**Keywords:** PCL–SBA-15 nanocomposites, real-time variable-temperature synchrotron measurements, confinement, mechanical behavior

## Abstract

A study of different nanocomposites based on poly(ε-caprolactone) (PCL) and mesoporous SBA-15 silica that were prepared by melt extrusion was carried out by analyzing the possible effect of this filler on the crystalline details of PCL, on its mechanical behavior, and on the eventual observation of the confinement of the polymeric chains within the hollow nanometric silica channels. Thus, simultaneous Small-Angle and Wide-Angle X-ray Scattering (SAXS/WAXS) synchrotron experiments at variable temperature were performed on these PCL nanocomposites with different mesoporous silica contents. The importance of the morphological and structural features was assessed by the changes that were observed during the mechanical response of the final materials, which determined that the presence of mesoporous particles leads to a noticeable reinforcing effect.

## 1. Introduction

Polycaprolactone (PCL) is one of the most attractive and most commonly used biodegradable polymers [1]. This material belongs to the category of polyesters that are derived from petrochemical feedstocks, and its main features include hydrophobicity, low glass transition (T_g_) and melting temperatures (T_m_) at about −60 °C and 60 °C, respectively, as well as important chain flexibility and outstanding processability. Furthermore, it can be spun into fibers or blown films at temperatures under 200 °C without experiencing thermal degradation. PCL degrades through the hydrolysis of its ester linkages, showing a relatively slow degradation rate [1]. This latest aspect makes PCL suitable for the fabrication of long-term implant systems and for its use as a matrix in drugs delivery systems, primarily those for controlled-release devices with long working lifetimes (1–2 years). However, PCL suffers from certain shortcomings when it is intended to be used in the tissue engineering field, including its aforementioned slow degradation rate, low cell adhesion and poor mechanical properties, which tends to be experienced under load-bearing conditions. Advanced manufacturing technologies such as electrospinning or 3D printing [2,3] and its blending it with other polymers [4,5,6,7,8,9] or incorporation of stiffer materials (fillers or fibers) [10,11,12,13] can promote the improvement of either its bioactivity or its mechanical response, enabling it to be used for bone tissue engineering applications.

Ordered mesoporous silicas emerged in the 1990s. MCM-41 and SBA-15 are the most well-known members, with both exhibiting hexagonal arrangements of uniformly sized cylindrical pores, a narrow pore size distribution, and a large surface area [14,15]. The diameter of the pores that are found in SBA-15 particles are larger than those that are found in MCM-41, with the former typically showing pore size diameters of 7–9 nm, and the latest demonstrating typical pore sizes of 3 nm. Because of their tailor-made pore shapes and sizes, these materials have become particularly important in applications that are based on molecular recognition, such as selective catalysis, molecular sieving, chemical sensing, and precise adsorption or in applications where they act as drug carriers. Furthermore, the incorporation of these ordered and hollow mesostructures as a minority component into neat polymers becomes a very attractive approach that can be used to achieve hybrid polymeric-based materials. In fact, they have been used as hosts and reactors in various polymerization reactions [16,17,18,19,20,21], where they also acted as catalyst carriers and as a reinforcing material [22,23,24,25]. In addition, these pristine mesoporous silicas are capable of chemical medication, which supposes a versatile range of methodologies that can be implemented to easily attain tailored functionalized polymeric composites [26,27] with fine-tuned performance characteristics.

Polymers may become confined in the pores during the polymerization or during their further processing if the macromolecular chains are able to be allocated in the nanometric spaces that exist in mesoporous silicas. This partial presence of a polymer within those hollow nanospaces may result in a more intimate phase affinity in the resulting composite, contributing to improved interface adhesion and leading to a significant enhancement in the ultimate properties, mainly the mechanical ones, since mesoporous silicas can act as a filler [18,22,23,24,26,27].

This research aims to prepare composites that are based on PCL and SBA-15 particles at different contents through extrusion in order to learn the effect that pristine mesoporous particles exert on the polymeric crystalline structure, thermal behavior, and mechanical response. Moreover, the presence of PCL chains within the nanometric pores of SBA-15 will be also evaluated. Extrusion was selected since it is a cost-effective and an environmentally friendly transformation protocol that does not involve the use of a solvent. Accordingly, the novelty of this research consists of proposing a sustainable strategy that can be implemented to improve mechanical performance of PCL-based materials at temperatures higher than room temperature by taking advantage of incorporating a more rigid component that allows the additional possibility of including the PCL chains confined within the regular nanometric arrangement of these materials. Thus, the influence of SBA-15 on the crystalline details of PCL will be assessed by X-ray diffraction, specifically with real-time variable-temperature X-ray scattering at both small angles (SAXS) and wide angles (WAXS). Differential scanning calorimetry (DSC) and SAXS experiments will be employed to verify eventual confinement occurs in the obtained materials. Finally, the ultimate mechanical performance will be examined by stress–strain tests.

## 2. Materials and Methods

### 2.1. Materials and Chemicals

A commercially available polycaprolactone (purchased from Sigma-Aldrich) with an average molar mass (M_n_) of 80,000 g/mol and a density of 1.145 g/cm^3^ was used as a polymeric matrix in the present research. SBA-15 particles were also purchased from Sigma-Aldrich (specific surface area, S_BET_ = 517 m^2^/g; total pore volume, V_t_ = 0.83 cm^3^/g; average mesopore diameter [28], D_p_ = 6.25 nm) and were used as received.

### 2.2. Nano Composite and Film Preparation

Composites with different contents of SBA-15 particles (3, 6, and 9% in weight, which were labeled as PCL-SBA3, PCL-SBA6, and PCL-SBA9, respectively) were processed by melt extrusion in a corotating twin-screw microextruder (Rondol). Both the polymer and SBA-15 were dried prior to extrusion. The PCL was placed in an oven at 50 °C for 20 min and was then dried under vacuum conditions at 50 °C for 20 h. The SBA-15 particles were dried under vacuum conditions at 100 °C for 24 h. In the extruder, a screw temperature profile of 100, 105, 110, 120, and 110 °C was used from the hopper to the die, where the length-to-diameter ratio was 20:1. Then, films were obtained by compression molding at 120 °C and at 30 bar for 6 minutes in a hot-plate Collin press. Afterwards, a cooling process was applied to the different materials from their molten state to room temperature for 4 min at the relatively fast rate of around 80 °C/min and at a pressure of 30 bar.

### 2.3. Transmission Electron Microscopy

Measurements were performed at room temperature under a 200 kV JEM-2100 JEOL microscope. The particles were dispersed in acetone in an ultrasonic bath for 5 min and were then deposited in a holder prior to observation.

### 2.4. Scanning Electron Microscopy

Images were attained using S-8000 Hitachi equipment at room temperature in different cryo-fractured sections of the composites with distinct mesoporous contents. These thin sections, which were around 40 nm in size, were cut by means of cryo-ultramicrotomy (Leica EM UC6) at −120 °C and were deposited in a holder.

### 2.5. Thermogravimetric Analysis

Thermogravimetric analysis (TGA) was performed in using the Q500 equipment from TA Instruments under either a nitrogen or air atmosphere and at a heating rate of 10 °C/min. The degradation temperatures of the distinct materials were determined as well as the exact amount of SBA-15 that had been incorporated into the composites prepared by extrusion, which was estimated as an average of the values that were obtained from the two environments.

### 2.6. Differential Scanning Calorimetry

Calorimetric analyses were carried out in a TA Instruments Q100 calorimeter that was connected to a cooling system and that had been calibrated with different standards. The sample weights were around 6 mg. A temperature interval ranging from −80 to 100 °C was studied at a heating rate of 20 °C/min. To determine the crystallinity of the samples, a value of 135 J/g was considered for the melting enthalpy of the 100% crystalline PCL [29,30]. Errors in the temperature determination, enthalpy calculation, and the crystallinity were estimated at ±0.5 °C, ±4 J/g, and ±0.04 units, respectively.

### 2.7. X-ray Experiments with Synchrotron Radiation

Simultaneous real-time variable-temperature SAXS/WAXS experiments were carried out using synchrotron radiation in the beamline BL11-NCD-SWEET at ALBA (Cerdanyola del Valles, Barcelona, Spain) at a fixed wavelength of 0.1 nm. A Pilatus detector was used for the SAXS (off beam, at a distance of 296 cm from sample) experiments, and a Rayonix one was used for the WAXS (at about 14.6 cm from sample, and a tilt angle of around 29 degrees) experiments. A Linkam Unit, which was connected to a liquid nitrogen cooling system, was employed for temperature control. The spacing calibration was determined by means of silver behenate and using Cr_2_O_3_ standards. The initial 2D X-ray images were converted into 1D diffractograms as function of the inverse scattering vector, *s* = 1/*d* = 2 sin θ/λ, using pyFAI python code (ESRF) that had been modified by the ALBA beamline staff. Film samples of around 5 × 5 × 0.2 mm were used for the synchrotron analysis.

### 2.8. Mechanical Behavior by Means of Stress–Strain Tests

Nominal stress–strain tests were performed in an MTS Q-Test Elite dynamometer with a load-cell of 100 N at a temperature of 25 °C and at a rate of 10 mm/min. The specimens that were used for these experiments were punched out from the polymer films. The dimensions of these strips were 15 mm in length, 1.9 mm in width, and around 0.20 mm in thickness. A minimum of six different strips were stretched until fracture at a given specimen.

## 3. Results and Discussion

### 3.1. Morphological Characteristics

**Figure 1:**
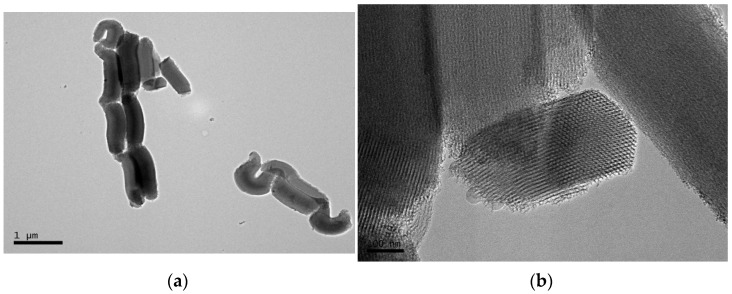
TEM images of SBA-15 particles at scale bars of (**a**) 1 μm and (**b**) 100 nm.

Details of the mesoporous SBA-15 silica were visible through the use of transmission electron microscopy (TEM), while the dispersion of SBA-15 particles within the PCL matrix was evaluated by high-resolution field emission scanning electron microscopy (FESEM). Figure 1a shows a TEM micrograph for the particles in the mesoporous SBA-15 silica that was used in this investigation. These particles display a vermicular and elongated shape with an approximate size of 350 nm in width and 0.9 μm in length. The size that is exhibited by this commercial mesoporous silica corresponds with the one that was previously described in the literature for mesoporous SBA-15 particles that had been synthesized in a laboratory [22]. Its magnification, shown in Figure 1b, depicts the interior particle morphology, which is made up of a well-defined, uniform, and ordered channel structure with hexagonal arrangements. 

**Figure 2:**
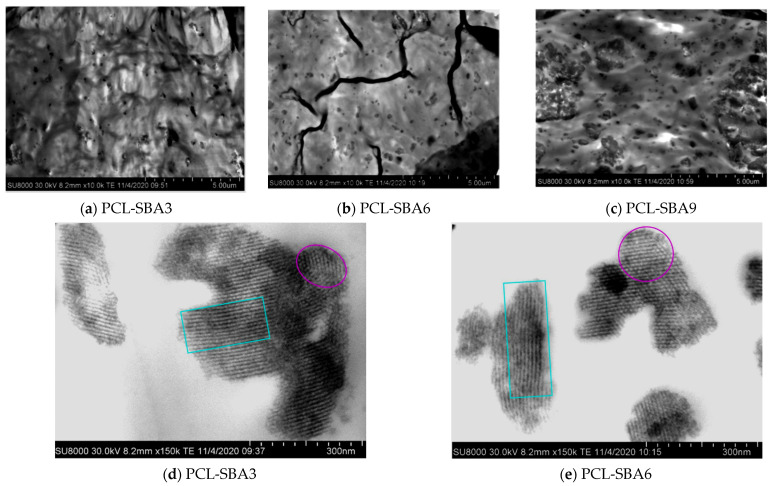
FESEM micrographs for the different composites: (**a**) PCL-SBA3; (**b**) PCL-SBA6; and (**c**) PCL-SBA9, at a scale bar of 5 μm. FESEM micrographs showing individual SBA-15 particles for different composites: (**d**) PCL-SBA3 and (**e**) PCL-SBA6, at a scale bar of 300 nm.

Figure 2 shows FESEM micrographs for the distinct composites based on PCL and several amounts of SBA-15 particles. The SBA-15 silica demonstrates a clear uniform dispersion in the pictures, and an evident increase in the number of SBA-15 particles is also observed, as their content is increased in the final composite (see precise compositions determined from TGA results in Table 1).

The non-existence of inorganic domains that are bulky in size is noticed across the materials that were fabricated with different contents of SBA-15. However, the formation of particles aggregates is evident, as the amount of silica in the material increases and is more apparent in the PCL-SBA9 composite, as depicted in the picture in Figure 2c.

The well-defined channel structure and the common hexagonal arrangement that is exhibited by these mesoporous SBA-15 particles are maintained when they are embedded in the PCL matrix of the different composites. Figure 2d,e undoubtedly display these features for the PCL-SBA3 and PCL-SBA6 composites. That ordering is also preserved in the composite with the highest content of mesoporous SBA-15, something that will be commented upon in more depth later. This indicates that at the mesoscale, regularity is not changed by the shear forces that are applied during extrusion, the protocol that was used for the preparation of the PCL-based materials in this study.

### 3.2. Thermal Stability

Mesoporous silicas have also sometimes been used as catalysts for thermal decomposition. This effect was found by Marcilla et al. [31] when degradation caused by TGA was studied in polyethylene (PE) under N_2_ in the presence and absence of mesoporous MCM-41. Other authors [32] have shown the efficiency of mesoporous alumino-silicate MCM-41 as a promoter of the polyolefin degradation into liquid fuels. This role as a promoter of decomposition was also found when MCM-41 silica was used as a catalyst carrier and filler for in situ polymerized PE-based composites that employed either neat mesoporous particles or those decorated with undecenoic acid or silanes [18,26,33,34].

**Figure 3:**
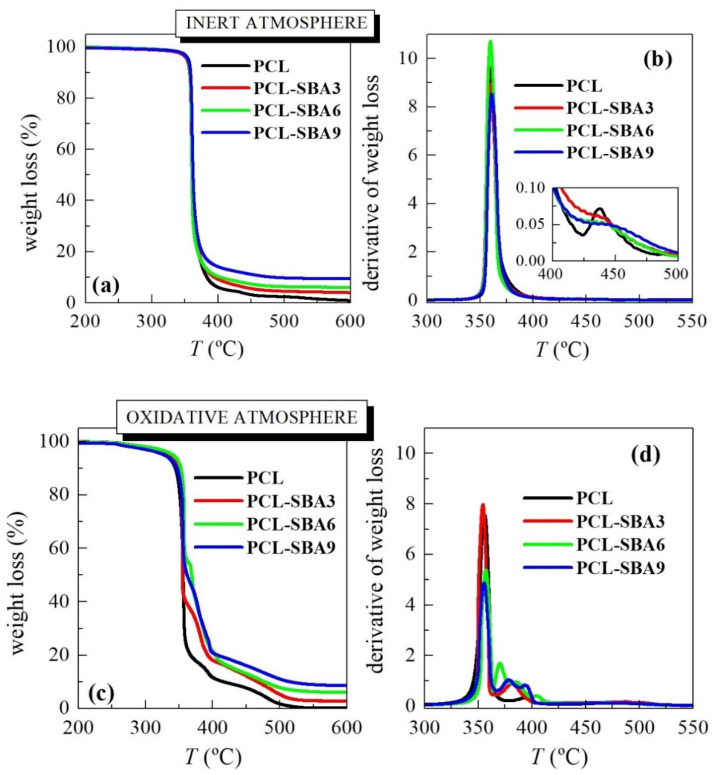
TGA curves either under inert atmosphere (**a**) and their derivatives (**b**) or in an oxidant environment (**c**) and their derivatives (**d**) for the neat PCL and its composites with SBA-15 at different contents prepared by melt extrusion.

Figure 3 shows the temperature dependence of PCL weight loss under either inert or oxidative conditions in these composites. The inert TGA curve (see Figure 3a) displays one main degradation process in the pristine PCL, with an inflection point at around 360 °C (see data in Table 1), which is where most of the weight loss (approximately 95%) occurs. Nevertheless, a careful examination of the DTGA trace (see inset in Figure 3b) highlights another minor consecutive mechanism that takes place at a higher temperature. By studying the nature of the gases that were produced through the entire thermal degradation process and under the inert environment allowed a two-stage degradation mechanism to be proposed [35,36]. The first process involved a statistical rupture of the polyester chains via an ester pyrolysis reaction. The produced gases were identified as H_2_O, CO_2_, and 5-hexenoic acid. The second step led to the formation of ɛ-caprolactone (cyclic monomer) as result of an unzipping depolymerization process.

If experiments are performed in an inert atmosphere, then thermal PCL degradability is not considerably affected by the incorporation of SBA-15 particles. The presence of mesoporous silica does not change the location at which most of the weight loss takes place (see values under the two atmospheres in Table 1) although the process that is related to unzipping depolymerization of PCL becomes a shoulder, and it is increased in the composites compared to in the pristine polymer. PCL degradation is affected by other parameters, such as polyester molar mass and the nature of the PCL end groups [35]. An increase in the former provokes a significant drop in the degradation rate. This behavior can be explained by the statistical chain cleavage that is triggered by the pyrolysis reaction during the first degradation process.

Figure 3c shows the temperature dependence of the PCL weight loss under an air environment. The behavior is more complex than that seen under the inert atmosphere. Thus, weight loss takes place in several stages, something that is clearly noticeable in the TGA and DTGA curves, since now PCL degradation occurs via both thermolysis and oxidation, i.e., through thermo-oxidation. The process that involves the highest amount of weight loss is not considerably affected by the presence of mesoporous SBA-15, as deduced from the T_max_ listed in Table 1. Its effect is more evident in the value of T_10%_. This is slightly shifted to higher temperatures in the composites, indicating that SBA-15 slightly postpones the beginning of PCL weight loss. All of these results indicate that the presence of SBA-15 particles in a PCL matrix does not exert a catalytic effect in these composites, contrary to the case of previous observations, where polyethylene was used as a matrix and where MCM-41 was used as the mesoporous silica [18,26,31,32,33,34]. Furthermore, the TGA curves that were obtained under both environments show that PCL degrades at an analogous range of temperatures independently of the atmosphere that is used.

### 3.3. Phase Transitions, Crystalline Characteristics and Confinement of PCL Chains

**Figure 4:**
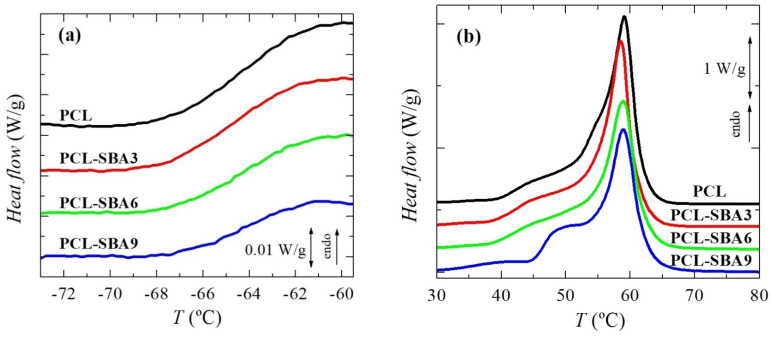
DSC curves (normalized to the actual PCL amount at a given material) for the neat PCL and its composites prepared by melt extrusion: (**a**) in the range of glass transition and (**b**) in the melting range. Curves have been shifted for the sake of clarity.

To analyze the effect of incorporating the mesoporous silica in the PCL transitions on the first heating run, the different transitions were evaluated separately, in the order of increasing temperatures: the glass transition temperature and the melting process temperature, as well as their corresponding characteristic temperatures, T_g_ and T_m_. Figure 4a shows the temperature range for neat PCL and its composites in the T_g_ interval, with this transition being related to the amorphous regions. These curves, which have been normalized to the actual PCL amount, display that the intensity of this transition is dependent on the silica content in the different composite samples. This could indicate that the quantity of PCL that is present in the amorphous regions decreased as a result of the silica particle incorporation, i.e., the crystallinity increased due to the presence of SBA-15 particles; however, the results listed in Table 2 do not confirm this hypothesis. On the other hand, this finding could be also ascribed to a hindrance in the amorphous mobility caused by mesoporous silica incorporation. The location remains almost constant, with only slight displacement being observed at higher temperatures in the samples with the highest SBA-15 contents.

Figure 4b shows the DSC curves in the temperature range for the melting of the PCL crystallites in the neat homopolymer and the composites during the first heating stage. A complex melting process is exhibited by the PCL in the pristine polymer [9,30] and in all of the composites. Thus, distinct endothermic events are noticeable, which can be ascribed to the existence of several processes. These multiple stages could be associated with the thermal history that was imposed during their processing, implying a fast cooling rate from the melt stage that leads to thin and imperfect crystals that are able to become thicker and more perfect via annealing at room temperature during the DSC run. The main melting peak is, however, rather analogous in all of these samples, as the size and amount of the major PCL crystallites is not affected by the incorporation of SBA-15, which can be deduced from the crystallinity values after they have been normalized to the actual PCL amount in the different materials, as listed in Table 2.

Regarding the secondary melting events, important differences can be observed in the interval ranging from 40 to 53 °C. Two different processes are considered to be present in this interval. The first one is the annealing of the initially imperfect crystals at room temperature [37,38,39,40]. The as-processed films were maintained for 2 days at ambient conditions before the DSC experiments, and during that time, the imperfect crystallites were able to thicken, thus increasing their melting temperature.

The second process is the eventual melting of the PCL crystals that are confined inside the SBA-15 pores, as those crystals are much thinner than the ones located in the outer surface of the mesoporous particles. Therefore, the melting temperature of such small crystals has to be significantly lower than those for the PCL crystals outside of the channels. The reason for this can be found by considering the Gibbs–Thomson equation. A simplified equation is used [41,42,43] in the case of “regular” lamellar crystals, but a more general version [44,45] is necessary for the thin crystals that are confined in the SBA-15 channels, which supposedly present very low values in their lateral size because of the pore diameters. As a consequence, the corresponding melting temperatures are considerably lower.

Obviously, confinement cannot exist in neat PCL, so the melting curve for this sample only includes the secondary process that is associated with the annealing that occurs at room temperature. Therefore, the eventual confinement that takes place in the composites can be approximately determined by subtracting the DSC profile of neat PCL from those of the composites. This is just an approximation since there may be differences in the thickening of the crystals by effect of the silica. For instance, the main crystal size is slightly dependent on the SBA-15 content (see below).

Previously, DSC experiments have been proven to demonstrate the existence of crystallites that are confined inside porous materials: in organic solvents within controlled pore glasses [46], in polyethylene in cylindrical nanoporous alumina [45], and in semicrystalline polyolefins within mesoporous silica [18,22,23,24,26,27,47,48], through the appearance of an additional minor endotherm, indicating a decrease in the melting point (and a reduced enthalpy). The sensitivity of this technique together with the multiple small melting processes that take place in PCL processed under these conditions currently only allow the observation that something different is taking place at the highest SBA-15 content, which is the PCL-SBA9 sample. The actual demonstration of confinement for PCL crystallites will be evaluated later by real-time variable-temperature small-angle X-ray scattering (SAXS) measurements with synchrotron radiation since they have turned out to be a valuable means to reveal the presence of polypropylene (PP) chains within the SBA-15 channels in nanocomposites that have been prepared by in situ polymerization and melt extrusion [24,48,49]. A thorough analysis performed from 0.095 to 0.13 nm^−1^ in the *s* scale, where the first order of the hexagonal arrangement of the SBA-15 particles is observed, allows the determination of the existence or absence of crystallites that are growing within the nanometric pores.

The overall degree of PCL crystallinity can be also determined from the DSC curves. As mentioned before, the results that were obtained during the first melting process, which were reported in Table 2, show that incorporating mesoporous silica does not change the PCL crystallization capability and that crystallinity remains rather constant in these films, which are processed by applying a fast cooling rate after the melt and then keeping the films at room temperature.

**Figure 5:**
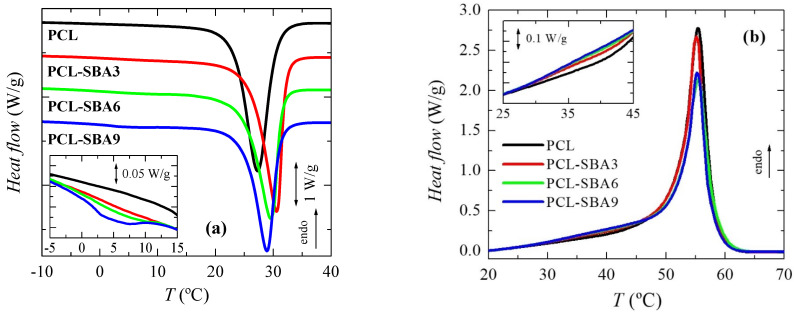
DSC curves corresponding to (**a**) cooling and (**b**) second heating run for the pristine PCL and its composites with SBA-15 particles. Curves have been shifted in the left plot for the sake of clarity.

This simultaneous existence in the first melting scan of the annealing process and the eventual melting of the confined PCL crystals does not occur in the subsequent cooling and second melting processes. Thus, the curves that were attained for the cooling process are represented in Figure 5a. An exothermic main event is clearly noticeable since these PCL-based materials are semicrystalline. Furthermore, it can be observed that the mesoporous SBA-15 particles exert an evident nucleating effect during PCL crystallization, and its location is shifted to a higher temperature in the composites. This easier capability of PCL to crystallize is more significant in the composite with the lowest silica content, this showing the highest T_c_ value. An increase in the SBA-15 composition leads to a progressive decrease in T_c_, which can probably be ascribed to the increase in size of the inorganic domains; however, the T_c_ in the composites is always superior to that found in pristine PCL (see data in Table 2).

In addition to this main exothermic event, another small crystallization process is observed in the composites from 0 to 10 °C, as seen in the inset of Figure 5a. This is more evident in the PCL-SBA9 material, and it almost goes undetected in PCL-SBA3. The pure PCL does not show any event in this temperature range. This process implies the formation of constrained crystallites, and it appears at lower temperatures. Since the pristine PCL does not show it, the only crystals that can be developed in these composite materials compared to in neat homopolymer are those from the PCL chains that are located within the nanometric SBA-15 channels. This point will be discussed in more detail along with results of the real-time temperature-variable SAXS experiments.

The PCL crystallinity was also estimated from these DSC curves, as listed in Table 2. A clear reduction in the crystallinity is clearly observed compared with that determined from the as-processed films. In spite of the DSC cooling rate is slower than that used for film preparation, 20 °C/min instead of at approximately 80 °C/min, and it should lead to thicker crystals, the difference is associated with the fact that the samples from the as-processed films were maintained, as mentioned before, at room temperature for 2 days before they underwent DSC testing, and during that time, the crystallites were able to undergo several melting–recrystallization processes and were able to increase in number, allowing crystallinity to increase. Under these conditions, the incorporation of mesoporous silica seems to result in a tiny diminishment in the PCL crystallinity within the experimental error.

Figure 5b depicts the DSC curves that were observed during the second heating run after PCL crystallization at 20 °C/min. The behavior is now simpler, without the presence of an annealing process being noted during the initial scan. The second heating process is initiated just after cooling finishes, and then the crystalline entities do not have enough time to develop more perfect crystallites. In addition, these crystals that are grown under 20 °C/min are thicker. Accordingly, melting–recrystallization processes are not noticeable in this second heating run, and the main T_m_ values are shifted to slightly lower temperatures. Its location seems to be independent of SBA-15 particle incorporation. The absence of those melting–recrystallization processes results in the crystallinity being lower than that deduced from the first melting runs. As the mesoporous silica content increases, the crystallinity is slightly reduced. The only effect of the presence of SBA-15 is seen in the interval that ranges between 30 and 45 °C, which is associated with the melting of the PCL crystals that are confined in the nanometric SBA-15 mesostructure. Evidence of confinement during DSC crystallization and during the second heating process was previously observed for high density PE (HDPE) [27] but not in the case of ultrahigh molecular weight PE (UHMWPE) [22,23,25,47] or extruded PP [48,49].

Much more detailed information can be deduced from real-time variable-temperature synchrotron experiments in the small-angle region, as aforementioned. The presence of PCL chains within the SBA-15 channel means that there is a certain amount of PCL within the constrained nanometric spaces and, accordingly, these systems could be called nanocomposites.

**Figure 6:**
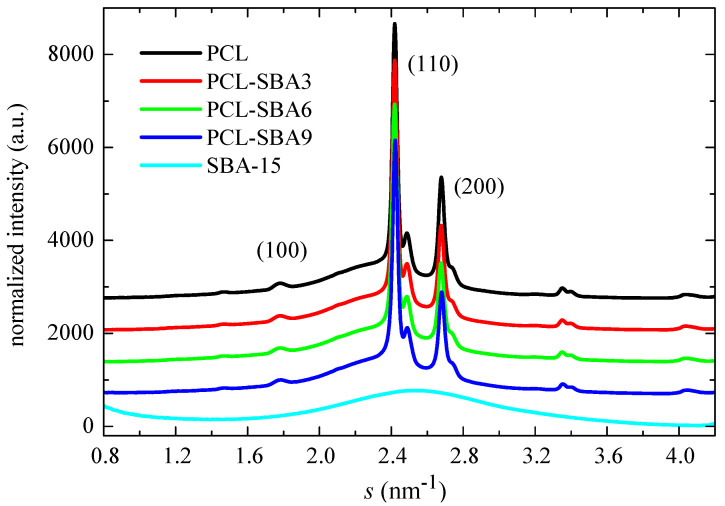
Wide angle profiles at room temperature, obtained with synchrotron radiation, for pristine PCL homopolymer, and SBA-15 particles together with those for the nanocomposites prepared by extrusion.

Figure 6 shows the WAXS profiles at room temperature for the pristine PCL, its composites, and the neat SBA-15 silica. The profiles were obtained through the use of synchrotron radiation. PCL typically crystallizes into an orthorhombic lattice [50]. There are no evident changes in the location of its characteristic diffractions ((100), (110), and (200) peaks) with the presence of mesoporous silica. The SBA-15 particles are, however, amorphous at a short-range, thus showing a wide halo, which is centered at the *s* value of 2.54 nm^−1^.

The degree of crystallinity can be also determined from wide-angle X-ray diffraction by comparison of the area under the crystalline peaks to the total scattered intensity when a two-phase model is considered [51]. For that, the corresponding amount of the amorphous halo must be subtracted. This amorphous profile was obtained from the real-time variable temperature WAXS experiments (see below). The crystallinity values are listed in Table 3. They do not vary much in the composites when compared to the values that are exhibited by the pristine PCL. A slightly decreasing trend is observed, and the data are within the experimental error. Moreover, these WAXS values are rather similar to those determined by DSC (see Table 2).

**Figure 7:**
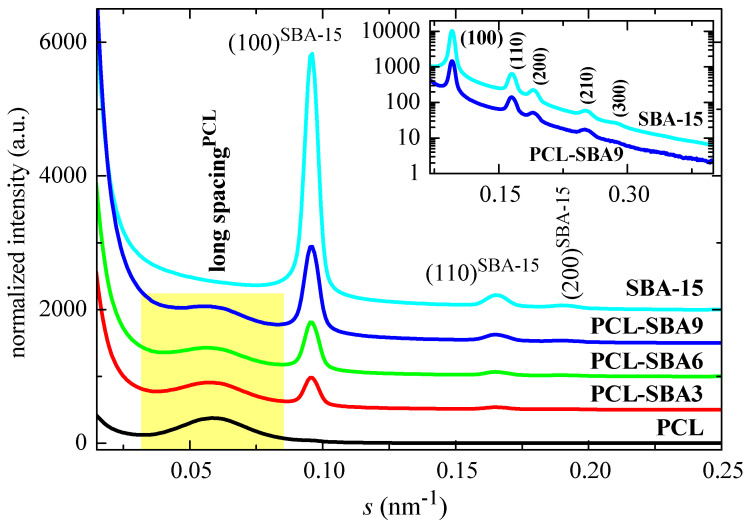
SAXS profiles at room temperature for the pristine PCL and the mesoporous SBA-15 as well as their composites.

Figure 7 shows the SAXS profiles for the neat PCL and mesoporous SBA-15 silica as well as its composites at room temperature. On one hand, it is clearly noticeable that the ordering of the mesoporous particles has been maintained throughout extrusion of the distinct composites, a fact that was found earlier from the FESEM pictures. Thus, the characteristic reflections of its hexagonal *p6mm* symmetry at (100), (110), (200), (210), and (300) [15,52] can be observed in the SAXS profiles. The inset in Figure 7 represents the profiles for SBA-15 and for the PLC-SBA9 composite in the logarithmic scale, making the smaller peaks more noticeable.

In addition to the diffractions ascribed to SBA-15, a broad peak is also evident in the region of low *s* values. This can be attributed to the variation in the electron density of the PCL matrix as consequence of its semicrystalline nature and its lamellar crystallites, i.e., it can be ascribed to its most probable long spacing. The values for the different samples are reported in Table 3. The presence of SBA-15 particles increases the PCL long spacing, as mesoporous content is raised in the composites. The most probable crystal size is also enlarged, increasing with the SBA-15 amount, in spite of a slightly decrease in the crystallinity values. These slightly thicker crystallites do not seem to affect the T_m_ of the first melting process and values remain almost constant (see Table 2). These similar T_m_ values can be ascribed to the existence of multiple melting–recrystallization processes during heating of the different materials.

**Figure 8:**
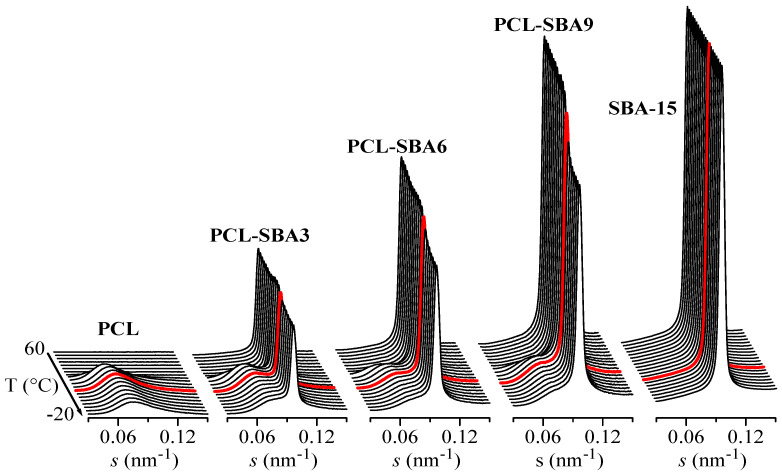
Lorentz-corrected synchrotron SAXS 1D diffractograms during the cooling from the melt at 20 °C/min of the different samples: neat PCL homopolymer and SBA-15 particles together with those for the three composites. Only one out every two frames were plotted for clarity. Highlighted in red: frame at T = 11 °C. The Y scale for SBA-15 is 10 times higher than for the other samples.

It has been previously reported that the existence of the polymeric chains that are confined in the channels of SBA-15 (and other mesoporous silicas) is clearly reflected in important variations in the intensity of the SAXS diffraction peaks of the silica, as observed in different polymeric composites [48,53,54,55,56]. These variations can be interpreted by considering that the intensity of SBA-15 diffraction not only depends on the amount of pore filling but also on the scattering contrast between the walls and the inside of the SBA-15 channels [57,58]. This is also the case for the present PCL composites. For instance, Figure 8 shows the Lorentz-corrected synchrotron SAXS 1D diffractograms that were obtained during the cooling from the melt at 20 °C/min of the neat PCL homopolymer and SBA-15 particles together with those for the three composites. A decrease in the SBA-15 diffraction intensity in the composites, which is centered at approximately 11 °C, is clearly observed and is directly proportional to the silica content in the composite. Additionally, it is important to note that the pristine SBA-15 sample does not show that decrease, with the intensity being constant throughout the entire temperature interval.

Therefore, the evident changes in the intensity of the SBA-15 peaks that are observed in Figure 8 for the composites can be interpreted as arising from variations in the scattering contrast before and after the crystallization of the PCL chains that are confined inside of the SBA-15 pores. These changes appear in the same temperature range as those that are observed for the secondary exotherm in the DSC curves of Figure 5a.

Moreover, Figure 8 also shows the long spacing of the PCL crystals at lower *s* values when the sample crystallizes from the melt, which occurs at approximately 30 °C for PCL as well as for the composites.

**Figure 9:**
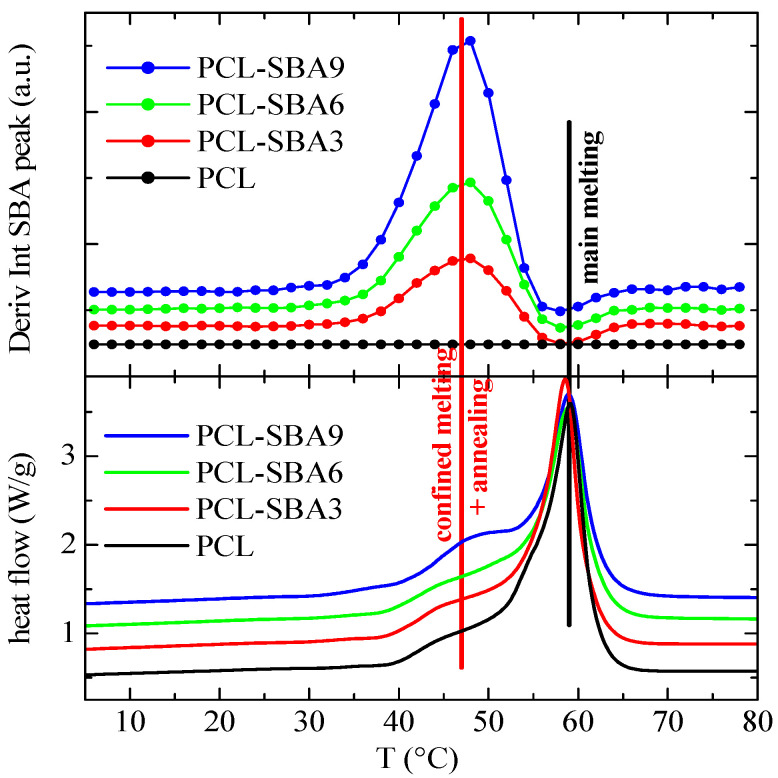
Variation in the temperature (**upper**) of the derivative of the intensity of the SBA-15 diffraction for the indicated samples during the first melting process, at 20 °C/min, compared to the corresponding DSC melting curves (**lower**). Derivative curves have been vertically shifted for clarity.

That change in the intensity of the SBA-15 peak also occurs during the first and second melting processes of the composites. In fact, the derivative of that intensity has been performed and plotted against temperature, allowing it to be compared to the corresponding DSC curves. For instance, Figure 9 shows the variation in that derivative with temperature in the case of the first melting process for the different composites (neat PCL has also been included, but its value is obviously always zero).

A comparison of the DSC melting curves allows to reach the important conclusion that the derivative of the intensity is only slightly sensitive to the main melting process, while considerably larger variations occur in the region of the secondary endotherms, which can be interpreted as being connected to the melting processes of the PCL crystals that are confined in the SBA-15 channels. Moreover, the magnitude of the derivative maxima in the composites is approximately proportional to their actual mesoporous silica content.

**Figure 10:**
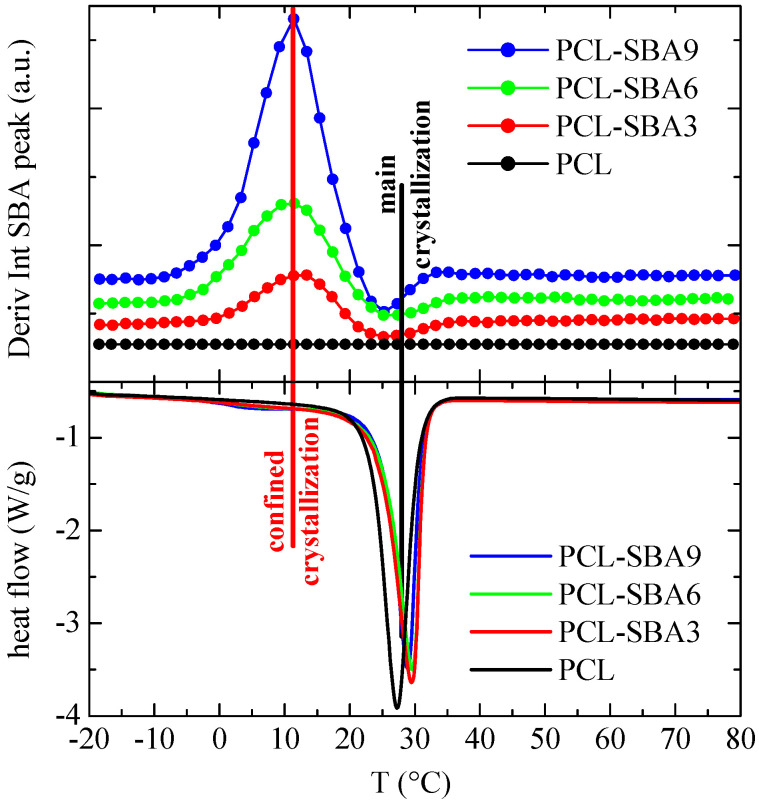
Variation with temperature (upper) of the derivative of the intensity of the SBA-15 diffraction for the indicated samples during the cooling from the melt, at 20 °C/min, compared with the corresponding DSC crystallization curves (lower). Derivative curves have been vertically shifted, for clarity.

The corresponding results after cooling from the melt are displayed in Figure 10. As before, a comparison with the DSC cooling curves indicates that the derivative of the intensity is a little sensitive to the main crystallization intensity, which occurs at around 30 °C, while variations are considerably higher in the region of the secondary exotherms, which are associated with crystallization of the PCL chains that are confined in the SBA-15 channels.

**Figure 11:**
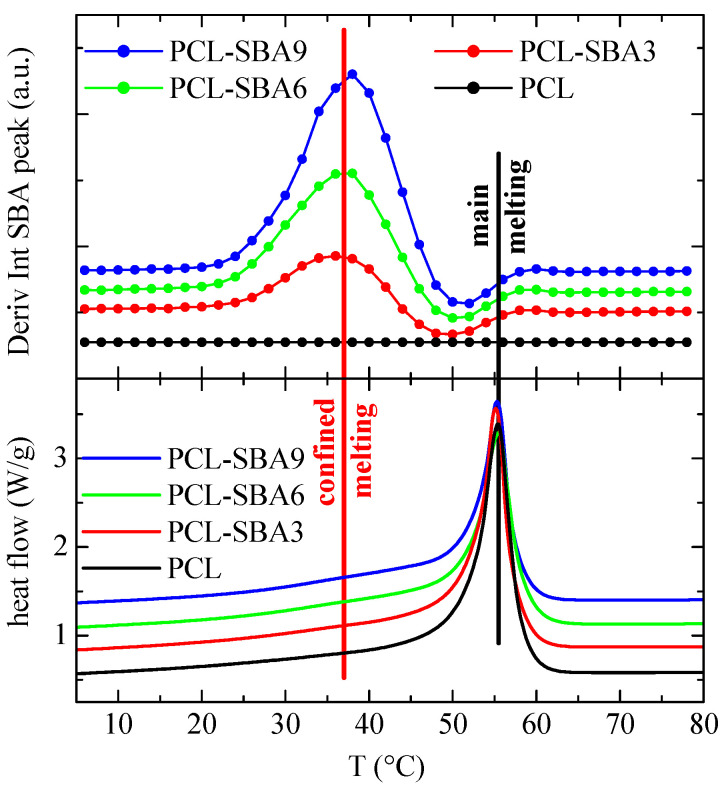
Variation in the temperature (upper) of the derivative of the intensity of the SBA-15 diffraction for the indicated samples during the second melting, at 20 °C/min, compared to the corresponding DSC melting curves (lower). Curves have been vertically shifted for clarity.

Finally, Figure 11 shows the variation in the temperature of the derivative of the SBA-15 diffraction intensity for the subsequent second melting process. Now, the variations that occur in the region of the secondary endotherms (confined melting) appear to be at a temperature that is somewhat lower than it is for the first melting process, which also happens to be the case for the DSC results. In fact, the confined melting occurs at around 47 °C during the first melting process, while it appears to take place at 38 °C for the second melting process. It, therefore, seems that by keeping the materials at room temperature, not only the “regular” PCL crystals outside of and surrounding the SBA-15 particles undergo annealing processes, but the ones that are confined inside of the silica channels do as well. This leads to a drop in the melting temperature of around 12 °C in the first case (Figure 9), while it amounts to about 18 °C during the second melting process (Figure 11). Additionally, this displacement is about 18 °C for the crystallization upon cooling (Figure 10).

Therefore, the previous results show the relevance of real-time variable-temperature SAXS experiments for the determination of the presence of polymeric crystals that are confined within the channels of the SBA-15 mesoporous silica.

**Figure 12:**
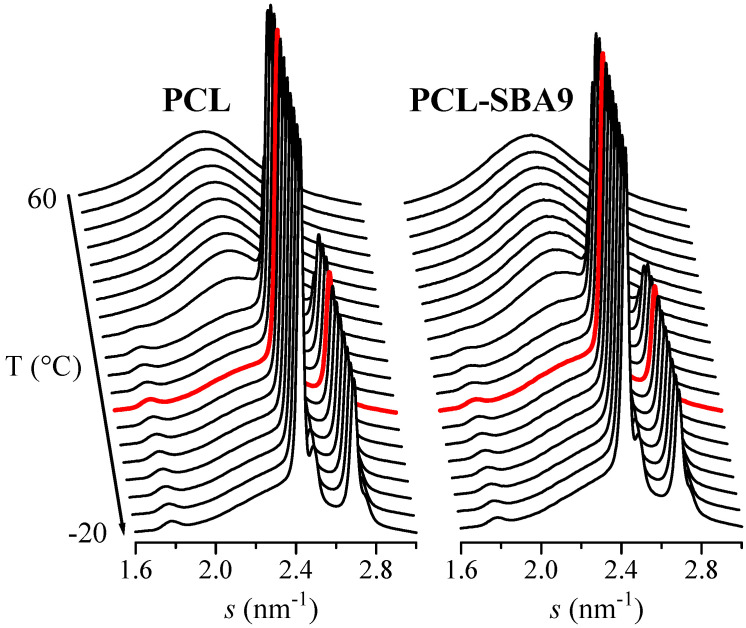
Synchrotron WAXS 1D diffractograms obtained during the cooling from the melt at 20 °C/min for neat PCL (left) and for the composite PCL-SBA9 (right). Only one out every two frames were plotted for clarity. Highlighted in red: frame at T = 11 °C.

On the other hand, the synchrotron WAXS 1D diffractograms that were attained during the cooling process after the melt at 20 °C/min for neat PCL and for the composite PCL-SBA9 are displayed in Figure 12. No information about confinement can be deduced from these WAXS profiles. Nevertheless, these diffractograms show that the main crystallization phase occurs at around 30 °C, which is in accordance with the DSC results. The temperature for this primary ordering is around 20 degrees higher than the one for the confined crystallization process that takes place inside the silica channels, which appears at around 11 °C, as previously seen in Figure 10.

### 3.4. Mechanical Properties

**Figure 13:**
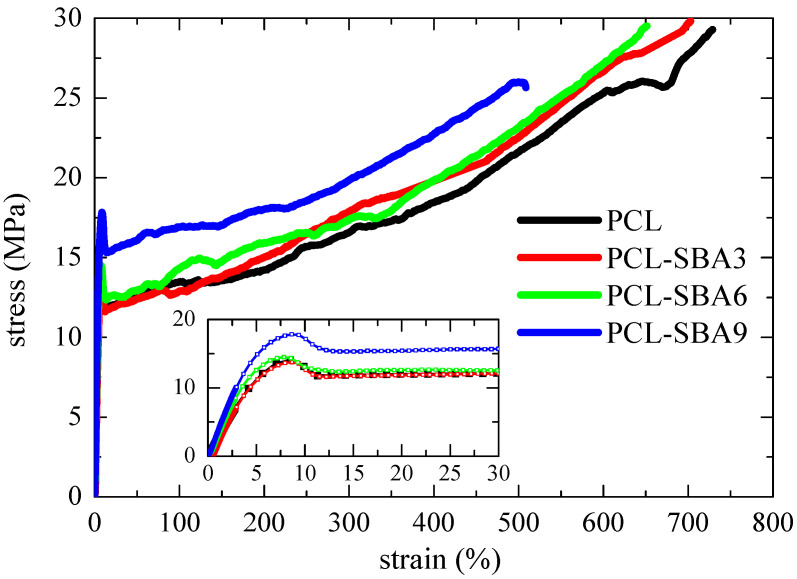
Stress–strain behavior of pristine PCL and its composites, which were prepared by extrusion with SBA-15 particles.

Figure 13 shows the stress–strain curves for the pure PCL and the different composites. The behavior that can be deduced from the nominal stress–strain curves for all of these specimens are characteristic of ductile polymeric materials. The engineering stress–strain curves display three distinct regions: initially, the stress rises as strain does in a linear dependence that allows Young´s modulus, E, to be determined. After this initial stage, an evident yield point is observed (see inset in Figure 13) followed by a narrow region where the stress is kept rather constant. Finally, a third region where another increase in the stress is observed, resulting in considerable strain-hardening that is related to the stress-induced orientation of the polymeric PCL chains being observed. Thus, the stress–strain curves of these samples are characterized by the formation of a neck during the deformation process, which was also confirmed during the direct observations that were made throughout the stretching process. On the other hand, the narrow necking propagation stage also indicates that these materials are relatively soft.

**Figure 14:**
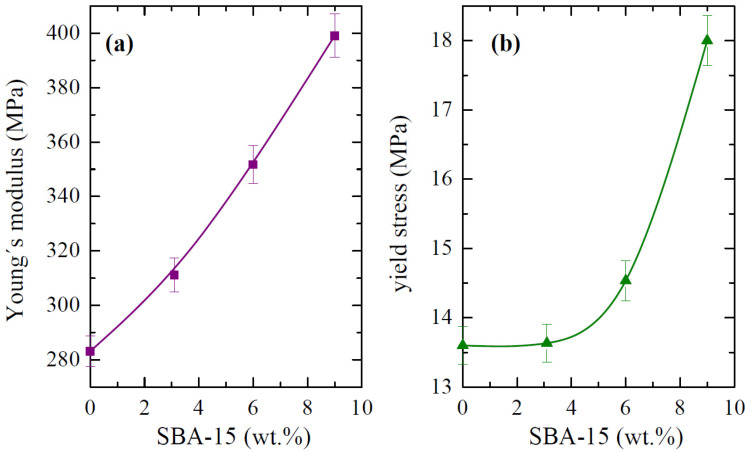
(**a**) Modulus values, E, and (**b**) yield stress, σ_Y_, both of which were deduced from stress–strain tests for the PCL homopolymer and composites with different SBA-15 contents.

The main parameters that were achieved from the curves that are depicted in Figure 13, Young´s modulus (E) and yield stress (σ_Y_), are represented in Figure 14. Regarding the E values, the incorporation of the silica particles leads to more rigid materials, and the dependence on the content is almost linear. Accordingly, the E values are at any filler content higher than that attained for the pristine PCL. In fact, PCL-SBA9 shows a value that is 40 % higher than for the neat PCL. At similar loading contents, this increase is superior to that found in nanocomposites based on polypropylene [49,56] but is analogous to the one observed when polyethylene is used as the matrix [22,59].

There are three possible explanations for this behavior in the studied PCL materials. On one hand, silica is stiffer than polymeric PCL. Thus, the inclusion of silica, regardless of the amount, should be expected to increase the elastic modulus proportionally to the amount of silica that is added. SBA-15 particles play a reinforcing role. Secondly, although crystallinity remained almost constant in the different materials, the crystallite size increases proportionally to the amount of mesoporous silica that is in the structure. Consequently, the PCL matrix in the nanocomposites is stiffer than it is in neat PCL. Finally, the rather good dispersion of SBA-15 particles within the PCL, the presence of PCL chains within the SBA-15 pores, and their crystallization capability in these confined nanospaces can contribute to improved interface adhesion, resulting in enhanced ultimate mechanical properties.

In relation to the yielding point, effect of the yielding stress on the SBA-15 content is somehow different to that exhibited by the E values. This could be associated with the fact that different contributions other than those that are exclusively related to rigidity (presence of fillers, polymer crystallinity and crystal size, etcetera) exert an effect when the plastic domain is initiated. Figure 14 clearly shows that no important variations in the absolute σ_Y_ values are observed between PCL and PCL-SBA3 in spite of an increase being noticed in the stiffness values. This could be ascribed to the fact that all of these materials behave in a ductile manner and possibly due to the fact that the amount of filler at that content is not high. However, the PCL-SBA6 and PCL-SBA9 composites exhibit considerably larger σ_Y_ values.

Other very interesting features are related to the strain at that break and the tensile strength. PCL shows the highest value since it is the softest material that was studied. The values decrease as the SBA-15 content increases, but PCL-SBA9 is able to be deformed up to a strain of 500 %. Its rigidity has been improved remarkably, but at rupture, these characteristics did not show a noticeable decrease. Concerning the tensile strength, the average values were determined to be at about 30 MPa for PCL, PCL-SBA3, and PCL-SBA6, and for PCL-SBA9, the tensile strength decreases to 26 MPa.

Furthermore, the stress–strain response demonstrated good reproducibility in terms of the shape of deformation process as well as in the mechanical magnitudes that were derived from these experiments for the different strips that were stretched for a given sample. This feature could be once again ascribed to an optimal SBA-15 dispersion within the PCL, as shown by the FESEM images in Figure 2.

## 4. Conclusions

Composites that are based on PCL and different amounts of mesoporous SBA-15 silica were prepared by means of melt extrusion. The well-defined hexagonal arrangement that is exhibited by pristine SBA-15 was found to be maintained in the particles that were embedded within the PCL matrix in the resultant composites, as shown by the FESEM pictures and the results of the SAXS experiments. Moreover, the SBA-15 particles demonstrate a rather homogenous distribution within the PCL matrix at the different contents.

The presence of an endothermic event in the first DSC melting curves, which take place at the temperature interval ranging from 40 to 53 °C, indicates the preliminary development of small crystals that are accommodated in the interior space of the nanometric SBA-15 channels. This means that there are confined PCL chains in the SBA-15 pores. This was also observed in the subsequent cooling and second melting experiments, where a secondary exotherm or endotherm, respectively, appeared. They were approximately proportional to the SBA-15 content in the composites.

The real-time variable-temperature SAXS experiments were found to be very important instrument for determining the presence of the PCL crystals that were confined within the channels of the mesoporous SBA-15 silica. Thus, the existence of those confined PCL chains can be clearly deduced from the important variations in the intensity of the main SAXS diffraction that is seen in the silica. Derivatives of this intensity are only slightly sensitive to the main melting process (or crystallization), while considerably higher variations occur in the regions that contain the confined entities.

The incorporation of SBA-15 particles does not demonstrate a large effect on the PCL thermal degradation processes under either inert or air atmospheres, and neither the location of T_g_ and main T_m_ nor the crystallinity values that were deduced from either the DSC or WAXS experiments. Nevertheless, the PCL crystallites became slightly thicker as the amount of mesoporous silica increased in the final material. This feature, together with the higher stiffness of the SBA-15 particles, is responsible for the remarkable reinforcement effect that was observed in the composites. Accordingly, Young´s modulus increased significantly as the SBA-15 content raised. Moreover, in terms of the break behavior, the rupture strain and tensile strength were suitably maintained.

The incorporation of SBA-15 seems to be an appropriate and useful strategy that can be used to improve the mechanical performance of PCL. This could allow this type of composites to be used in a more diverse range of applications that require stiffer materials and an enhanced mechanical response.

## Figures and Tables

**Table 1:** TGA results under nitrogen and air atmospheres for neat PCL and its composites prepared by melt extrusion: temperature of a loss weight of 10% (T_10%_) and temperature at the maximum (T_max_) together with the SBA-15 wt.% at 600 °C and its average.

Sample	Average SBA-15 wt.%	*Inert Atmosphere*			*Oxidative Atmosphere*		
		T_10%_ (°C)	T_max_ (°C)	SBA-15 wt.%	T_10%_ (°C)	T_max_ (°C)	SBA-15 wt.%
PCL	0	359.0	360.0	0	344.5	356.0	0
PCL-SBA3	3.3	357.5	359.5	3.8	350.5	354.5	2.8
PCL-SBA6	6.0	358.0	359.5	6.0	353.0	357.5	6.0
PCL-SBA9	9.0	358.5	361.0	9.3	347.0	355.5	8.6

**Table 2:** Average SBA-15 wt.% estimated from TGA measurements and DSC results: glass transition (T_g_)—calculated from the first heating run—, melting (T_m_), and crystallization (T_c_) temperatures, and overall crystallinity (normalized to the actual PCL content in the material) for the first melting (f_c_^m1^_NORM_), crystallization (f_c_^C^_NORM_), and second melting (f_c_^m1^_NORM_) processes.

Sample	SBA-15 wt.%	T_g_ (°C)	T_m1_ (°C)	f_c_^m1^_NORM_	T_c_ (°C)	f_c_^C^_NORM_	T_m2_ (°C)	f_c_^m2^_NORM_
PCL	0	−64.5	59.0	0.51	27.5	0.42	55.5	0.42
PCL-SBA3	3.3	−64.5	58.5	0.50	30.5	0.41	55.0	0.41
PCL-SBA6	6.0	−64.0	59.0	0.50	29.5	0.41	55.5	0.41
PCL-SBA9	9.0	−64.0	59.0	0.51	29.0	0.40	55.5	0.40

**Table 3:** Characteristics of the PCL crystalline phase for the pristine polymer and the different composites: f_c_^PCL^_WAXS_ (crystallinity degree determined by WAXS at room temperature); L^PCL^_SAXS_ (long spacing estimated by SAXS at room temperature); and l_c_ (most probable crystal size calculated assuming a two-phase model: l_c_ = L^PCL^_SAXS_ ⋅ f_c_^PCL^_WAXS_).

Sample	SBA-15 wt.%	f_c_^PCL^_WAXS_	L^PCL^_SAXS_ (nm)	l_c_ (nm)
PCL	0	0.54	17.1	9.2
PCL-SBA3	3.3	0.53	17.6	9.3
PCL-SBA6	6.0	0.52	17.8	9.3
PCL-SBA9	9.0	0.52	18.1	9.4

Standard errors: f_c_^NORM^_WAXS_ ± 4%; L^SAXS^ and l_c_ ± 0.3 nm.

## Data Availability

Data are contained within the article.
